# Patient selection strategies for deep inspiration breath-hold in left-sided breast cancer: a review of current evidence and future directions

**DOI:** 10.3389/fonc.2026.1949600

**Published:** 2026-10-13

**Authors:** Xiangshang Sun, Shixiong Huang, Xing Zhou, Borong Zhou, Zhiyan Wang, Lingrong Luo, Qionghui Zhou, Biao Zeng, Qianxi Ni

**Affiliations:** 1 Department of Radiation Oncology, The Affiliated Cancer Hospital of Xiangya School of Medicine, Central South University/Hunan Cancer Hospital, Changsha, China; 2 Department of Chest Radiation Oncology, The Affiliated Cancer Hospital of Xiangya School of Medicine, Central South University/Hunan Cancer Hospital, Changsha, China

**Keywords:** artificial intelligence, breast cancer, cardiac sparing, deep inspiration breath-hold, patient selection

## Abstract

With accumulating evidence confirming that postoperative adjuvant radiotherapy improves survival outcomes in patients with left-sided breast cancer, the long-term cardiotoxicity risks associated with radiotherapy have attracted widespread attention. The deep inspiration breath hold (DIBH) technique increases the distance between the heart and the breast target, effectively reducing radiation dose to the heart and coronary arteries. However, the resource-intensive nature of DIBH and substantial heterogeneity in therapeutic benefits among patients pose a major clinical challenge: how to accurately and efficiently identify patients suitable for DIBH. This narrative review summarizes current evidence regarding DIBH patient selection, and integrates available strategies with clinical workflows to construct a three-tiered decision-making framework encompassing feasibility assessment, indication evaluation, and clinical benefit assessment. Given the existing gaps in high-level prospective clinical evidence, three primary research directions are proposed: (1) conducting large-scale multi-center prospective cohort studies to validate the generalizability and clinical practicality of selection strategies; (2) developing an individualized normal tissue complication probability (NTCP) model integrating multi-dimensional risk factors to guide precise decision-making; and (3) implementing prospective clinical trials of artificial intelligence (AI)-assisted selection platforms to accelerate the clinical translation of intelligent selection tools. This review synthesizes current evidence on DIBH patient selection, constructs a hierarchical decision framework to provide a reference for clinical practice, and delineates directions for future research.

## 1 Introduction: cardiac sparing challenges in radiotherapy for left-sided breast cancer

Radiotherapy, as a critical component of comprehensive treatment for breast cancer, plays an important role in reducing local recurrence rates. However, the core challenge of radiotherapy for left-sided breast cancer is the substantially higher risk of radiation-induced cardiac injury relative to right-sided breast cancer radiotherapy, particularly in patients undergoing adjuvant radiotherapy after mastectomy or breast-conserving surgery. Epidemiological studies indicate that for every 1 Gy increase in the mean heart dose (MHD), the risk of major coronary artery events increases by 7.4% (1). For patients with left-sided breast cancer, due to the anatomical proximity of the chest wall target to the heart, even with the modern intensity-modulated radiotherapy (IMRT) technique under free-breathing (FB) conditions, the heart, especially the left anterior descending artery (LAD), may still receive a non-negligible radiation dose. With the prolonged survival of patients with breast cancer, late cardiovascular complications induced by radiotherapy have become a key factor affecting their long-term quality of life.

To address this challenge, various cardiac sparing techniques have emerged, including conformal shielding of the heart region using multi-leaf collimators (2, 3), prone position radiotherapy (4–6), and proton therapy (7, 8). DIBH (9–11) provides an effective solution for reducing cardiac radiation dose by increasing the distance between the breast and the heart. The clinical implementation of DIBH requires additional medical resources, such as surface-guided imaging systems, respiratory gating devices, and extended treatment duration. Therefore, establishing precise patient selection criteria to identify individuals at high risk of substantial cardiac exposure and those who may derive significant benefit from DIBH is crucial. This paper reviews the available selection strategies and proposes a hierarchical decision framework to illustrate a progressive decision pathway from feasibility assessment to indication evaluation and finally to clinical benefit assessment, providing a structured reference for clinical practice. The search strategy for this narrative review involved searching PubMed, Web of Science, and CNKI from database inception to December 30, 2025, using keywords such as “DIBH”, “patient selection”, “cardiac sparing”, and “left-sided breast cancer”. Studies were considered relevant if they investigated DIBH feasibility, patient selection criteria, anatomical or dosimetric indicators, biological indicators, or AI-assisted selection strategies in patients receiving left-sided breast radiotherapy. Studies without direct relevance to patient selection or cardiac-sparing decision-making were excluded. Reference lists of relevant studies were also reviewed to identify additional literature.

## 2 Cardiac sparing mechanisms and dosimetric advantages of DIBH

### 2.1 Anatomical basis and physiological mechanisms

DIBH increases the distance between the heart and the breast target, effectively reducing the radiation dose to the heart and coronary arteries. Notably, the breathing pattern significantly affects the degree of organ displacement (12). Abdominal deep inspiration breath-hold (A-DIBH) and thoracic deep inspiration breath-hold (T-DIBH) represent two distinct breathing patterns. A-DIBH primarily involves diaphragmatic descent and abdominal expansion, resulting in greater caudal displacement of the heart and more pronounced cardiac sparing. In contrast, T-DIBH relies mainly on thoracic expansion with relatively less diaphragmatic movement, resulting in a weaker cardiac displacement effect. Previous studies have shown that A-DIBH reduces the mean heart dose by 60%, compared with 46% for T-DIBH, indicating that A-DIBH yields a superior cardiac-sparing effect.

### 2.2 Clinical evidence of dosimetric benefits

The reduction in the cardiac radiation dose is the most direct clinical benefit of DIBH. Multiple studies have confirmed that DIBH can reduce the MHD by 26.2%-75% (13, 14). The observed benefit variations are influenced primarily by differences in patient surgical types (breast-conserving therapy (BCT) vs. postmastectomy radiotherapy (PMRT)), target volume, and treatment techniques. Additionally, lung tissue sparing represents another major advantage of DIBH. Although lung tissue is less sensitive to radiation than the heart, radiation pneumonitis remains a common complication of breast cancer radiotherapy. Previous studies indicate that DIBH can reduce the mean dose to the left lung by 16.3% to 19.6%, decreasing the percentage of left lung volume receiving 20 Gy (V_20_) from 19.6% to 15.3% (15) (P<0.001). Subgroup analysis of this study revealed that, compared with patients who underwent BCT, those who received PMRT experienced a more pronounced reduction in V_20_ after DIBH was adopted.

### 2.3 Clinical translation of dosimetric benefits

The dosimetric enhancements resulting from DIBH directly translate into a decrease in NTCP. Studies have shown that for patients in the low-risk group (free-breathing mean heart dose, FB-MHD < 4Gy), the NTCP for long-term cardiac mortality was 0.08%± 0.07% under FB and 0.07%± 0.06% under DIBH, representing a relative reduction of 12.5%. For patients in the high-risk group (FB-MHD ≥ 4 Gy), the NTCP was 0.66%± 0.43% under FB and 0.57% ±0.38% under DIBH, representing a relative reduction of 13.6% (16). Based on the Framingham Coronary Heart Disease Risk Score model (17) and Darby’s cardiac dose-response relationship model (1), the median relative risk reduction for coronary artery disease over 10 years after adopting DIBH was 32% (range from 16.15% to 83.28%), with an absolute risk reduction of 1.48% (range from 0.25% to 7.95%) (18). These findings hold substantial significance for enhancing the long-term prognosis of young patients and those with concurrent cardiovascular risk factors.

In addition, improved target position stability during DIBH may reduce uncertainties caused by respiration, representing another important advantage of DIBH beyond cardiac dose reduction. However, whether this allows for PTV margin reduction depends on multiple factors, including immobilization, image guidance strategies, respiratory monitoring, and intra- or inter-fraction reproducibility. For example, in the institutional workflow reported by Yu et al. (19), DIBH allowed for a reduced PTV margin of 6–8 mm compared with 6–10 mm under FB conditions. Nevertheless, these values should be regarded as institution-specific examples rather than a universal DIBH recommendation. PTV margins should be individually determined and validated according to the setup accuracy, motion management strategies, image guidance protocols, and reproducibility of individual institution.

## 3 Selection criteria

DIBH patient selection should not be viewed as a single binary decision but rather as involving multiple clinical considerations across various stages of the clinical workflow. In this review, a hierarchical framework was constructed to systematically organize the available selection criteria in accordance with the clinical workflow. This framework presents a sequential decision-making process involving three dimensions: (1) technical/physiological feasibility, which assesses whether a patient can perform and reproducibly maintain DIBH; (2) clinical, anatomical, dosimetric indications, which evaluate whether FB treatment would result in clinically significant cardiac or coronary artery exposure; and (3) expected clinical benefit, which considers whether the anticipated dose reduction is likely to translate into a clinically meaningful risk reduction. A schematic decision framework for DIBH patient selection based on the clinical workflow is illustrated in **Figure 1**. The AI-assisted selection strategies can be flexibly deployed at various stages of the clinical workflow, and all of which are detailed in Section 3.4.

**Figure 1:**
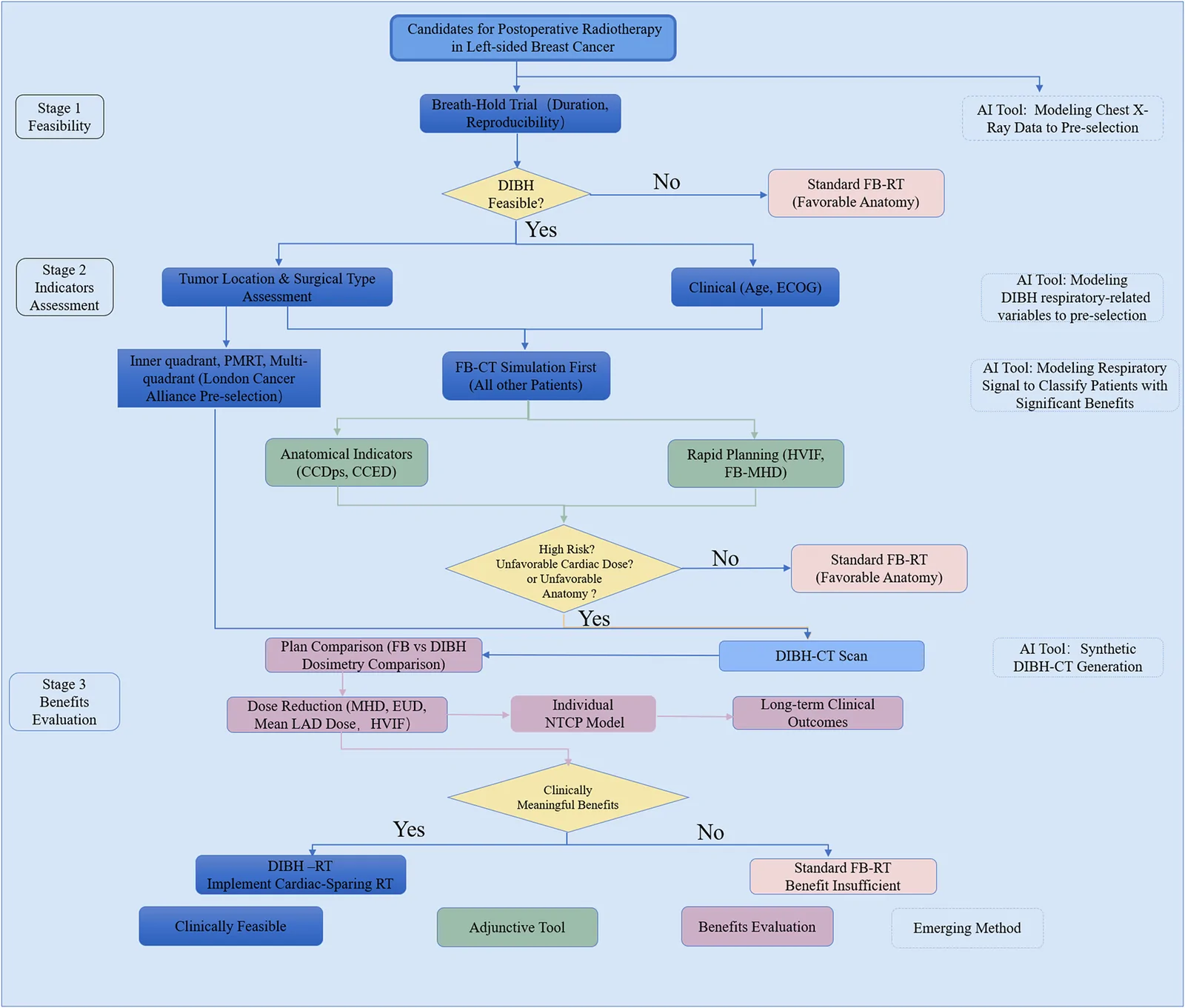
Schematic decision-making framework for DIBH patient selection based on clinical workflow. Color code denotes the evidence maturity level of each selection strategy. ECOG, Eastern Cooperative Oncology Group; DIBH, deep inspiration breath-hold; FB-RT, free-breathing radiotherapy; DIBH-RT, deep inspiration breath-hold radiotherapy; PMRT, post-mastectomy radiotherapy; CCDps, cardiac contact distance in the parasagittal plane; CCED, cardiac-chest Euclidean distance; HVIF, volume of the heart covered by 50% isodose line; FB-MHD, free-breathing mean heart dose; MHD, mean heart dose; EUD, equivalent uniform dose; LAD, left anterior descending artery; NTCP, normal tissue complication probability; AI, artificial intelligence.

### 3.1 Technical feasibility assessment

Feasibility assessment first determines whether a patient can perform and reproducibly maintain DIBH before addition imaging and treatment-planning resources are committed. Various respiratory and physiological characteristics have been investigated, including breath-hold duration, inspiratory amplitude, reproducibility, and pulmonary function.

Respiratory assessment provides a more direct evaluation of technical feasibility. Ahmad et al. (20) demonstrated that breath-hold duration and lower amplitude were associated with DIBH feasibility. Lower amplitude refers to the minimum stable amplitude the patient achieved across repeated breath-hold attempts; a higher value indicates that the patient maintained a deeper and more stable inspiratory level during the least favorable breath-hold attempt. Gaál et al. (21) reported a strong correlation between vital capacity and ipsilateral lung volume under DIBH conditions (r = 0.796, p = 0.002), whereas no statistically significant correlation between these variables was observed under free-breathing conditions (r = 0.451, p = 0.142). These findings suggest that direct breath-hold assessment and pulmonary function testing can provide useful information for evaluating technical feasibility, although the evidence for pulmonary function testing remains preliminary.

These approaches are clinically advantageous because they can be performed before CT simulation with relatively limited additional resources, and may avoid unnecessary DIBH-CT acquisitions and associated planning workloads in patients with clearly inadequate breath-hold performance. Direct assessment of breath-hold duration and amplitude is more closely related to the actual technical requirements of DIBH but requires respiratory monitoring and standardized assessment. Spirometry is objective and widely available in some centers, but current evidence supporting its routine use for DIBH selection is limited. Overall, pre-CT feasibility assessment should be considered a low-cost initial selection strategy, followed by clinical, anatomical, and dosimetric assessment to determine the indication for DIBH, and by subsequent evaluation of its expected clinical benefit.

### 3.2 Clinical, anatomical, and dosimetric indicators for DIBH patient selection

Once a patient is deemed technically feasible for DIBH, the next step is to determine whether DIBH is clinically indicated based on the potential cardiac exposure under free-breathing conditions. Clinical and treatment-related characteristics may enable early risk stratification with minimal resources, whereas anatomical indicators require CT-based geometric assessment. Dosimetric indicators directly assess potential cardiac exposure under free-breathing conditions, but require treatment planning and additional resources. Thus, these approaches play complementary roles in the clinical workflow, which progresses from early risk stratification to evaluation of the indication for DIBH based on potential cardiac exposure.

#### 3.2.1 Clinical and treatment-related characteristics

Age and functional status may provide simple clinical information for patient selection. Linge et al. (22) found that patients who underwent DIBH were younger and had better functional status compared with those treated with free-breathing. Tumor location and surgical type can also be assessed prior to CT simulation and may help identify patients with a higher likelihood of cardiac exposure, as illustrated by the London Cancer Alliance selection protocol (23). These factors primarily support practical risk stratification rather than direct assessment of DIBH feasibility.

#### 3.2.2 Cardiac anatomical indicators

Cardiac anatomical indicators primarily characterize the baseline geometric relationship between the heart and target or beam field, and may help identify patients who could derive significant benefit from DIBH. Several metrics have been proposed, including cardiac-chest wall contact distance (CCD), cardiac-chest Euclidean distance (CCED), cardiac risk index (CRI), heart volume inside the irradiation field (HVIF), and maximum heart in field (MHF). Although these metrics share a common overall objective, they differ significantly in measurement complexity and dependence on treatment planning. A schematic illustration of the measurement of key cardiac anatomical indicators is presented in **Figure 2**.

**Figure 2:**
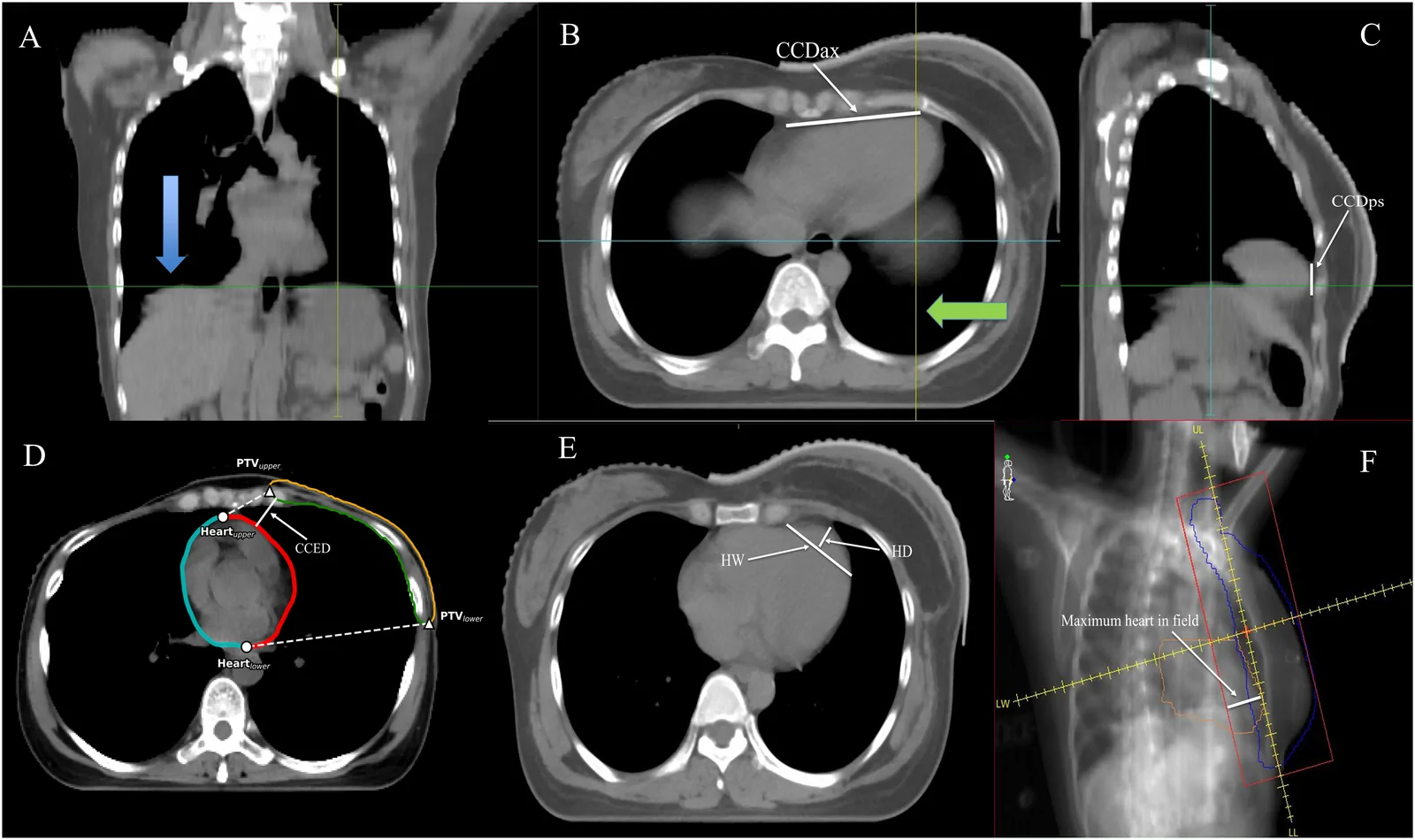
Schematic illustration of the measurement of key cardiac anatomical indicators. The top of the right diaphragm and the midpoint of the left hemithorax are landmarks for measurement, indicated by arrows in subfigures **(A, B)**. The measurement of CCDax in subfigure **(B)** and CCDps in subfigure **(C)** is indicated by a white line. **(D)** Demonstration of the measurement of the cardiac-to-chest Euclidean distance (CCED), which is defined as the shortest Euclidean distance between the anterior cardiac wall and the posterior chest wall. The axial plane shown is the plane in which this shortest Euclidean distance is measured across the entire three-dimensional space, rather than within a single two-dimensional plane. **(E)** Demonstration of the measurement of heart depth (HD) and heart width(HW). **(F)** Demonstration of the measurement of maximum heart in field (MHF) with a tangential beam’s-eye-view.

##### 3.2.2.1 Cardiac-chest wall contact distance

CCD quantifies the degree of contact between the heart and the chest wall and can be measured in either the parasagittal or axial plane. Parasagittal CCD (CCDps) is measured on the sagittal plane passing through the midpoint of the left hemithorax, while axial CCD (CCDax) is measured on the axial plane corresponding to the top of the diaphragm. Rochet et al. (24) and Cao et al. (25) reported that free-breathing CCDps was significantly associated with heart dose, whereas FB-CCDax showed no significant correlation with heart dose. These findings suggest that CCDps may capture clinically relevant baseline cardiac geometry more effectively than CCDax. Its primary advantage lies in its simplicity, and measurements can be obtained directly from routine CT images without requiring a treatment plan. However, its predictive value has been primarily validated in retrospective cohorts, and the inconsistent performance of different CCD definitions highlights the dependence of geometric surrogates on the specific measurement methodology.

##### 3.2.2.2 Cardiac-to-chest Euclidean distance

CCED is defined as the shortest Euclidean distance between the anterior cardiac wall and the posterior chest wall. Zhou et al. (16) reported that CCED exhibited an AUC of 0.83, and predicted FB-MHD ≥ 4 Gy with a sensitivity of 95% and a specificity of 70% at a study-specific cut-off value of 2.5 mm. Compared with a single-plane contact distance, CCED provides a more direct three-dimensional (3D) representation of the cardiac-chest wall proximity relationship, potentially offering greater anatomical discrimination. Similarly, CCED can be measured directly from routine CT images. Nevertheless, the reported cut-off values originate from specific study populations and endpoints and should not be interpreted as universally validated thresholds.

##### 3.2.2.3 Heart width, heart depth

Sung et al. (26) evaluated heart width (HW) and heart depth (HD) in 312 patients who underwent breast-conserving surgery and proposed the cardiac risk index (CRI), defined as the product of maximum HD and maximum HW. CRI demonstrated the highest discrimination ability for heart V_25_ (AUC = 0.969), with a sensitivity of 100% and a specificity of 88% at a study-specific cut-off of 14.58 cm². Although CRI integrates multiple geometric features into a single metric, its high reported performance was achieved within a single cohort for a specific dosimetric endpoint. Therefore, its clinical value remains to be confirmed in independent populations.

##### 3.2.2.4 Heart volume inside the irradiation field

HVIF quantifies the heart volume covered by the 50% prescription isodose line, thereby incorporating both cardiac anatomy and beam field geometry. Kim et al. (27) demonstrated a quantitative relationship between changes in HVIF (ΔHVIF) and reductions in MHD and mean LAD dose. Ferdinand et al. (28) further reported that study-specific ΔHVIF cut-offs of 6 cm³ and 13 cm³ were associated with MHD reductions of >20% and >50%, respectively. HVIF is more closely related to dosimetry but requires an initial treatment plan. Thus, it should be viewed as a planning-assisted selection indicator rather than a simple anatomical selection indicator. Its clinical usefulness may increase as automated planning and rapid dose calculation become more widespread.

##### 3.2.2.5 Maximum heart in field

MHF is defined as the point where the largest amount of heart was visible in the axial plane, extending to the posterior edge of the tangential field, and can be assessed directly on FB-CT scans. Browne et al. (29) reported that patients with an MHF of ≥ 0.7 cm experienced a significant reduction in MHD after DIBH. From a clinical implementation perspective, MHF is particularly attractive because it is simple, intuitive, and requires no 3D reconstruction or an initial treatment plan. However, its dependence on tangential field geometry limits its generalizability across different treatment techniques. Therefore, the reported threshold should be considered a study-specific cut-off, rather than a universal indication for DIBH.

Taken together, cardiac anatomical indicators represent a spectrum of increasing clinical complexity. Simple contact-distance indicators such as CCDps and MHF can be obtained relatively quickly from routine imaging, making them attractive for triage based on early images. Composite indicators such as CRI may provide greater discrimination but require more measurements and are less well validated. Planning-assisted indicators such as HVIF more directly reflect the interaction between cardiac anatomy and beam field, but they require additional planning resources. Currently, no single anatomical indicator has been sufficiently validated to serve as a universal selection criterion. Instead, the choice of indicator should depend on the clinical decision point, available resources, and whether further dosimetric assessment is required. Among all approaches, most reported thresholds remain study-specific and require external validation before being adopted as universal selection criteria. The anatomical indicators reported in these studies are summarized in **Table 1**.

**Table 1:** Summary of anatomical indicators for patient selection in DIBH radiotherapy.

Author	Anatomical metrics	Sample size	Patient population	Radiotherapy technique	Prediction endpoints	Reported cut-off value	Highlights
Rochet (24), Cao (25)	FB-CCDps	35 (Rochet); 67 (Cao)	PMRT+BCT (Rochet) Whole breast RT (Cao)	3D-CRT	MHD reduction	No fixed cut-off value	The FB-CCDps is positively and linearly correlated with the MHD (r = 0.6); it is also positively correlated with the reduction in MHD when DIBH is employed (r = 0.73). For every 1 cm increase in FB-CCDps, the reduction in MHD increases by 0.58 Gy when DIBH is employed.
Zhou (16)	CCED	53	PMRT	IMRT	FB-MHD ≥ 4 Gy	≤2.5 mm	The AUC of CCED for predicting FB-MHD ≥ 4 Gy is 0.83; it is an effective anatomical metric for predicting the MHD. At a reported threshold of 2.5 mm, CCED can be used to identify patients with FB-MHD ≥ 4 Gy with a sensitivity of 95% and a specificity of 70%.
Sung (26)	CRI=HDmax×HWmax	312	BCT	3D-CRT	Heart V_25_ ≥ 10%	≥14.58 cm^2^	The CRI has the highest area under the curve (AUC = 0.969) to predict heart V_25_. When the CRI is 14.58 cm^2^, the sensitivity of this metric can reach 100%, while the specificity is 88%.
Kim (27)	HVIF	97	Left-sided breast cancer	3D-CRT	MHD reduction	See linear equation	A linear regression equation between △HVIF and the dose reduction for heart and LAD was established. After adopting DIBH techniques, the reduction in MHD = 8.1 (cGy/cc) × △HVIF(cc) + 26.0 (cGy); the reduction in mean LAD dose = 81.6 (cGy/cc) × △HVIF(cc) + 109.1 (cGy).
Ferdinand (28)	△HVIF	31	PMRT+BCT	3D-CRT	Percentage reduction in MHD	“> 6cm^3^ or 13 cm^3^”	Using 6cm^3^ as the threshold for △HVIF, patients with a reduction ≥20% in MHD can be identified. Using 13cm^3^ as the threshold for △HVIF, patients with a reduction ≥50% in MHD can be identified.
Browne (29)	MHF	31	Left-sided breast cancer	3D-CRT	MHD reduction	>0.7 cm	When the MHF under FB is ≥ 0.7 cm, DIBH results in a negative correlation between △MHD and the MHF under FB. However, when the MHF under FB is < 0.7cm, this correlation no longer exists.
Chang (30)	DIBH(ILV)/ FB(ILV)	21	PMRT+BCT	IMRT	MHD reduction	≥1.8	When the ipsilateral lung volume of the patient is ≥ 950 cc, or the total lung volume is ≥ 2200 cc, the dose to the heart and LAD will significantly decrease. When the ILV ratio between DIBH and FB exceeds 1.8, the MHD decreases by 48%, and the mean LAD dose decreases by 56%.

#### 3.2.3 Parameters related to changes in lung volume

**Lung volume difference**: Lung-volume-related indicators provide a complementary measure of the respiratory expansion achieved during DIBH. Chang et al. (30) reported that both ipsilateral and total lung volumes were inversely correlated with cardiac and LAD doses, and identified study-specific volume thresholds associated with greater cardiac dose reduction. An increase in DIBH-to-FB ipsilateral lung volume ratio was also associated with significant reductions in MHD and mean LAD dose. Unlike purely anatomical metrics, lung-volume parameters reflect both thoracic anatomy and the physiological response to deep inspiration. However, these indicators typically require paired FB and DIBH images, making them less suitable for initial pre-CT selection. Currently available thresholds should be regarded as study-specific predictors rather than established clinical standards.

#### 3.2.4 Dosimetry-based selection strategies

Dosimetry-based selection represents a more treatment-specific approach because it directly quantifies cardiac exposure under free-breathing. Among available indicators, FB-MHD is particularly intuitive because it reflects the baseline cardiac dose that DIBH may reduce. In a cohort of 53 patients, Zhou et al. (16) used 4 Gy as a study-specific FB-MHD threshold and found that patients with FB-MHD ≥ 4 Gy exhibited greater cardiac dose reduction and a more pronounced decrease in estimated NTCP compared with those with lower baseline exposure. The primary advantage of FB-MHD lies in its direct dosimetric relevance, whereas its principal limitation is the requirement for an initial treatment plan on FB-CT. Therefore, it is more resource-intensive than anatomical selection and may be most useful when the potential benefit of DIBH remains uncertain after initial assessment.

Rapid automated planning may alleviate this resource burden. Wang et al. (31) developed an automated workflow that generated a preliminary IMRT plan from FB-CT and identified patients with unfavorable cardiac exposure using cardiac V_50_/HVIF. Such approaches illustrate how automation may transform dosimetry-based selection from a time-consuming planning step into an almost instantaneous decision-support process. Nevertheless, the clinical utility of selection strategies based on automated planning depends on the accuracy of the preliminary plan, the robustness of the selected threshold, and the balance between reducing unnecessary DIBH imaging and avoiding the risk of false-negative classification.

Overall, dosimetric indicators are most closely linked to actual radiation exposure risk but require more resources than purely anatomical selection. Therefore, a pragmatic workflow might initially use simple clinical and anatomical indicators to identify patients requiring further assessment, followed by dosimetry-based assessment under FB when the potential benefit of DIBH remains uncertain.

### 3.3 Dosimetric and biological indicators of expected clinical benefit

The evaluation of clinical benefits extends beyond technical feasibility and anatomical or dosimetric assessment. The key question is whether the expected cardiac dose reduction is sufficiently large to translate into a clinically meaningful cardiac risk reduction. While comparing FB and DIBH plans provides the most direct assessment of potential benefit, its clinical significance depends on the baseline cardiac dose, the selected dosimetric endpoint, and individual cardiovascular risk. Therefore, dose reduction should be viewed as an important but insufficient surrogate indicator for long-term cardiovascular benefit.

NTCP-based models may help convert dose differences into predicted toxicity risks. However, these estimates are model-dependent and should not be interpreted as directly observed reductions in cardiovascular events or cardiac mortality. Thus, NTCP is best used as a complementary tool for comparative risk assessment rather than as a standalone selection criterion.

Biological indicators may provide additional patient-centered evidence. In a prospective study of 58 patients with left-sided breast cancer treated with breast-conserving surgery, Shen et al. (32) evaluated the dynamic changes in myocardial enzyme levels after radiotherapy. They found that the risk of subclinical myocardial injury was negligible for the subgroup of patients with baseline FB-MHD < 2 Gy, indicating that DIBH offers limited incremental benefit for this specific biological endpoint. Biological indicators may complement traditional anatomical and dosimetric indicators by providing additional evidence regarding subclinical myocardial injury, rather than by relying entirely on dosimetric indicators as an indirect surrogate for clinical benefit.

### 3.4 AI-assisted selection strategies

Generally, DIBH patient selection depends primarily on clinical experience, respiratory training performance, and subsequent dosimetric assessments, making the entire process complex and resource-intensive. Recently, AI and machine learning have shown great potential to optimize this process by predicting DIBH feasibility, estimating potential dosimetric benefits, and reducing unnecessary imaging and planning procedures. In this section, AI-assisted selection strategies are categorized based on prediction endpoints, evidence maturity, and validation methods.

Owing to the inherent flexibility of AI methodologies, current AI models can be applied across various stages of the clinical workflow. AI-assisted selection strategies can be classified into three categories. First, models based on basic patient characteristics and breath-hold parameters aim to predict DIBH feasibility. Chufal et al. (33) developed a gradient boosting ensemble model that incorporated parameters collected during the patient’s initial DIBH session (such as breath-hold amplitude, breath-hold duration, and consistency across different cycles), as well as basic clinical variables like age and body mass index (BMI). This model demonstrated excellent performance in predicting DIBH infeasibility (AUC = 0.83) and could potentially reduce unnecessary assessments by approximately 20%. Among the available approaches, the validation framework for this model is relatively mature because it underwent temporal validation. Nevertheless, external validation is still required.

Second, models based on respiratory waveforms attempt to predict patient-specific dosimetric benefits. Vendrame et al. (34) trained a bidirectional long short-term memory (BiLSTM) network using respiratory motion trajectories collected during CT simulation, with the reduction in maximum LAD dose as the prediction endpoint. Despite being limited by a small sample size, this approach enables “prediction during scanning” without the need to design two separate treatment plans. This study is currently in the internal validation stage, and its generalizability requires further investigation.

Third, image-based approaches may support earlier selection or reduce the need for additional DIBH imaging. Koide et al. (35) used standard-dose planning FB-CT images and a U-Net model to generate synthetic DIBH-CT images for cardiac dose evaluation. The results indicated that DIBH-CT generated by the synthetic method was highly consistent with actual scans in both geometric shape and dose distribution. This approach may avoid additional DIBH-CT acquisitions, thereby reducing cumulative imaging radiation exposure. However, it remains unproven whether this reduction in imaging exposure translates into a clinically significant decrease in radiation-induced secondary malignancy risk. This proof-of-concept study underwent internal validation, but its generalizability requires further investigation. A preliminary supplement abstract by Chufal et al. (36) relied solely on chest X-ray data collected under breath-hold conditions and modified a convolutional neural network (CNN) to predict DIBH suitability. The model achieved an accuracy of 80% in the test set. However, this preliminary supplement abstract requires peer review and external validation.

These studies demonstrate that AI-assisted selection strategies are evolving from predicting whether DIBH is feasible toward estimating whether DIBH is worth implementing. This distinction is clinically crucial because technical feasibility does not necessarily imply substantial cardiac benefit. Moreover, given differences in prediction endpoints, patient populations, and intended applications, it must be recognized that higher AUC values do not necessarily indicate better clinical utility. For predictive models intended for clinical application, calibration, sensitivity and specificity at clinically significant thresholds, false-negative results, and external validation are of equal importance. At present, most models remain limited by small sample sizes, institution-specific datasets, and insufficient external validation. Therefore, AI-assisted selection strategies should be viewed as decision-support tools to complement, rather than replace clinical assessment and individualized dosimetric evaluation. The predictive models described above are summarized in **Table 2**.

**Table 2:** Summary of AI-based models for patient selection in DIBH radiotherapy.

Author	Algorithm and data sources	Prediction endpoints	Verification types	Evidence maturity	Highlights
Chufal (33)	Algorithm: Gradient boosting ensemble model. Data: First-day training parameters (breath-hold amplitude, duration, consistency across different cycles) and basic clinical variables	Prediction of DIBH feasibility	Model development with temporal validation	Moderate maturity	Requires only first-day data, enabling early identification of unsuitable patients. Reduces subsequent multi-day assessment workload by 20%. Large sample size with temporal validation, supporting good robustness. Suitable for centers already equipped with DIBH training facilities and workflows.
Vendrame (34)	Algorithm: Bidirectional LSTM Data: Respiratory waveform signals acquired during CT simulation	Prediction of DIBH-related sparing of maximum LAD dose	Proof-of concept with internal validation	Early stage	Directly predicts dosimetric benefit. Enables “prediction during scanning”. Suitable for resource-constrained centers or as a preselection tool before formal DIBH-CT scans.
Chufal (36)	Algorithm: CNN model Data: Chest X-ray images obtained under breath-hold	Binary classification of DIBH suitability	Supplement abstract with internal validation	Supplement abstract with preliminary evidence	Uses low-cost, widely available X-ray data. Suitable for centers with limited facilities or high patient throughput.
Koide (35)	Algorithm: U-Net model Data: Standard-dose planning FB-CT acquired during free breathing	Prediction of heart dose via synthetic DIBH-CT to determine suitability	Proof-of concept with internal validation	Early stage	Eliminates the need for an additional true DIBH-CT scan. Potentially avoids an additional DIBH-CT acquisition and reduces cumulative imaging exposure. Suitable for centers concerned with radiation safety or with limited CT capacity for preselection.

## 4 Critical considerations

### 4.1 Limitations of the current evidence

Despite significant advances in several approaches for DIBH patient selection, the current evidence is constrained by methodological heterogeneity and insufficient external validation. Most studies are single-center retrospective analyses, and the findings may be influenced by local patient populations, radiotherapy techniques, and respiratory management protocols, limiting the generalizability of the selection strategies. Although Chufal et al. (33) performed temporal validation for their machine learning model, external validation is still explicitly listed as a task for future research.

Regarding selection criteria, a major limitation is the lack of standardized selection indicators and thresholds. Some studies concentrate on CT anatomical parameters, others emphasize breath-hold function assessment, while others develop multi-parameter models by integrating basic clinical information. Direct comparison among these studies is difficult, and there is no consensus on the optimal thresholds for patient selection. Even for commonly used indicators such as FB-MHD, certain studies recommend 4 Gy (16), whereas others suggest 3 Gy (23). These discrepancies may reflect differences in patient populations, treatment techniques, and prediction endpoints. Thus, the current evidence does not support universal cut-off values, and comparative validation of candidate thresholds remains to be conducted.

The most fundamental limitation lies in the absence of clinical endpoints. Almost all current studies have only evaluated reductions in MHD, LAD dose, and other cardiac dosimetric indicators. Although cardiac dose serves as an established predictor of cardiovascular risk, these indicators remain intermediate endpoints and cannot directly quantify the magnitude of clinical benefit derived from DIBH. Furthermore, the lack of patient-reported outcomes is a common shortcoming in current studies. DIBH not only offers dosimetric advantages but also involves subjective experiences, including discomfort during breath-hold and psychological stress due to prolonged treatment duration.

### 4.2 Limitations of the present review

This review also has several limitations. As a narrative review, the literature search and study selection procedures are not fully systematic, which may lead to selection bias. In addition, owing to significant heterogeneity among patient populations, radiotherapy techniques, selection indicators, and predictive endpoints, it is not possible to directly compare or quantitatively synthesize the reported thresholds and model performance. Moreover, the rapid development of AI-assisted tools may render some evidence outdated. Consequently, this review categorizes these studies according to their developmental stage to provide appropriate context for readers. These limitations should be taken into account when interpreting the conclusions of this review.

## 5 Future directions

### 5.1 Large-scale, multi-center prospective cohort study

Currently, most studies concerning DIBH patient selection are single-center retrospective designs, which limit the quality and generalizability of the available evidence. Large-scale, multi-center prospective cohort studies are needed in the future to validate current selection strategies across different patient populations, radiotherapy techniques, and clinical workflows. The BRAVE-HEART clinical trial (37) has taken an important step in this direction. This trial aims to record and quantify the cardioprotective effect of the active breathing coordinator-assisted DIBH in routine clinical practice, providing prospective evidence regarding the clinical efficacy, reproducibility, and practical implementation of DIBH for cardiac protection. Future studies should standardize criteria for patient enrollment, data collection, selection indicators, and validation endpoints, while incorporating both dosimetric and long-term clinical outcomes. In addition, the impact of different planning techniques, including three-dimensional conformal radiotherapy (3D-CRT), IMRT, and volumetric-modulated arc therapy (VMAT), should be considered, as differences in dose distribution may affect the relative benefit of DIBH. Such studies are essential for establishing more generalizable selection criteria.

### 5.2 Individualized NTCP model integrating multi-dimensional risks

Although mean heart dose is currently the most widely used predictor of cardiac toxicity, increasing evidence indicates that patients’ inherent baseline cardiovascular risk factors also significantly influence the absolute risk of radiation-induced cardiac toxicity. Future selection strategies should shift from a single dose threshold to personalized NTCP models that integrate patient characteristics, cardiovascular risk factors, and dose distribution parameters. Factors such as hypertension, diabetes, dyslipidemia, smoking history, and prior cardiac disease can be combined with substructure-specific dose to estimate the absolute difference in long-term cardiac risk between DIBH and FB. Prior work on incorporating dose modification factors into NTCP modelling for breast cancer proton therapy (38) offers a potential methodological reference. Such models not only help determine whether DIBH reduces dose, but also establish whether the expected dose reduction is clinically meaningful for specific patients.

### 5.3 Prospective clinical trials of AI-assisted selection platforms

Machine learning models have demonstrated predictive performance in DIBH patient selection, but the clinical implementation requires rigorous prospective evaluation. In the future, it is essential to design and conduct high-quality prospective randomized controlled trials or practical clinical trials to determine whether AI-assisted selection strategies improve the accuracy of identifying patients who may benefit from DIBH, reduce unnecessary training, imaging, and planning work, and enhance workflow efficiency without increasing false-negative rate.

The clinical trial design of AI-assisted selection platforms requires special attention to several key factors. First, the intended role of each model should be clearly defined, particularly whether it is used for positive selection or exclusion, as the required sensitivity and specificity may differ. Second, the stability of models under different MHD thresholds, clinical settings, and planning strategies should also be evaluated. Related studies have shown that the predictive performance of machine learning models varies with changes in MHD thresholds and parameter settings, with the decision tree model exhibiting greater robustness at lower thresholds (39). Moreover, developing interpretable AI models to clarify the basis of model decisions can enhance clinicians’ trust and acceptance. Third, when deploying an AI-assisted selection platform, integration with clinical workflows must be considered. The pre-selection strategy proposed by Wang et al. (40) is capable of evaluating DIBH suitability in 11.9 minutes, achieving efficient integration with clinical workflows. These advances provide a basis for further prospective evaluation, but their clinical utility has not yet been established.

## 6 Conclusion

The cardiac-sparing effect of DIBH in left-sided breast radiotherapy is supported by substantial evidence; however, accurately identifying patients who are both able to perform DIBH and likely to derive significant benefit from it remains a major clinical challenge. This review synthesizes current selection strategies and presents a hierarchical decision-making framework to illustrate a progressive decision pathway from feasibility assessment to indication evaluation, and then to clinical benefit assessment, providing evidence-based support for clinical practice. Nevertheless, the current evidence is limited by heterogeneous study quality, inconsistent prediction endpoints, and a lack of standardized methodologies, and the pathway from model development to clinical translation has not yet been established.

Future research should prioritize multi-center prospective validation of existing selection strategies, develop individualized NTCP models integrating anatomical, dosimetric, and cardiovascular risk factors, and conduct prospective evaluations of AI-assisted selection platforms. These efforts may shift DIBH patient selection from experience-based judgment toward more personalized and evidence-based decision-making, while clarifying which patients are most likely to derive clinically meaningful benefits from DIBH radiotherapy.
